# Phase‐Controlled Momentum Bandgaps in Dual‐Channel Dispersive Photonic Time Crystals

**DOI:** 10.1002/nap2.70344

**Published:** 2026-11-13

**Authors:** Hongzhi Chen, Keyu Jin, Yingjie Wu, Bingtao Gao, Hongsheng Chen, Lian Shen

**Affiliations:** ^1^ State Key Laboratory of Extreme Photonics and Instrumentation ZJU‐Hangzhou Global Scientific and Technological Innovation Center, Zhejiang University Hangzhou China; ^2^ College of Information Science and Electronic Engineering The Electromagnetics Academy at Zhejiang University, Zhejiang University Hangzhou China; ^3^ Zhejiang Key Laboratory of Intelligent Electromagnetic Control and Advanced Electronic Integration Jinhua Institute of Zhejiang University, Zhejiang University Jinhua China

**Keywords:** dispersive photonic time crystals, dual‐channel modulation, dynamic control gain and loss, momentum bandgaps

## Abstract

Dispersive photonic time crystals (PTCs) offer a versatile platform for manipulating light–matter interactions through temporal modulation of material responses, enabling dynamic control of optical gain and loss. Previous multi‐parameter temporal modulation studies provide additional degrees of freedom for engineering the band structure. Quantitatively characterizing momentum‐bandgap splitting and understanding the interplay between distinct modulation channels are essential for achieving precise control over Floquet coupling and amplification–attenuation dynamics. Here, we systematically investigate a Lorentz‐dispersive PTC with simultaneous modulation of the plasma frequency and damping factor. Using Floquet expansion, we investigate how these two modulation channels individually and jointly modify the band structure. We show that the modulation amplitudes and relative phase jointly determine the effective coupling between Floquet modes, enabling flexible control over the bandgap size and the imaginary part of the eigenfrequency. Furthermore, the competition between modulation‐induced amplification and intrinsic material damping allows the relative phase to control transitions between net temporal amplification and attenuation. Floquet spectral calculations and finite‐difference time‐domain simulations further reveal the corresponding phase‐dependent spectral and field‐evolution characteristics. These findings establish a quantitative analytical approach for multi‐parameter band structure engineering and open new opportunities for phase‐controlled optical gain and loss in time‐varying photonic systems.

## Graphical abstract

A relative phase can determine whether a lossy photonic time crystal amplifies or attenuates light. By simultaneously modulating the squared plasma frequency and damping, we quantify how interference between two Floquet coupling pathways reshapes momentum bandgaps and controls complex eigenfrequencies. Analytical theory and numerical simulations demonstrate a route toward phase‐programmable photonic gain–loss dynamics.

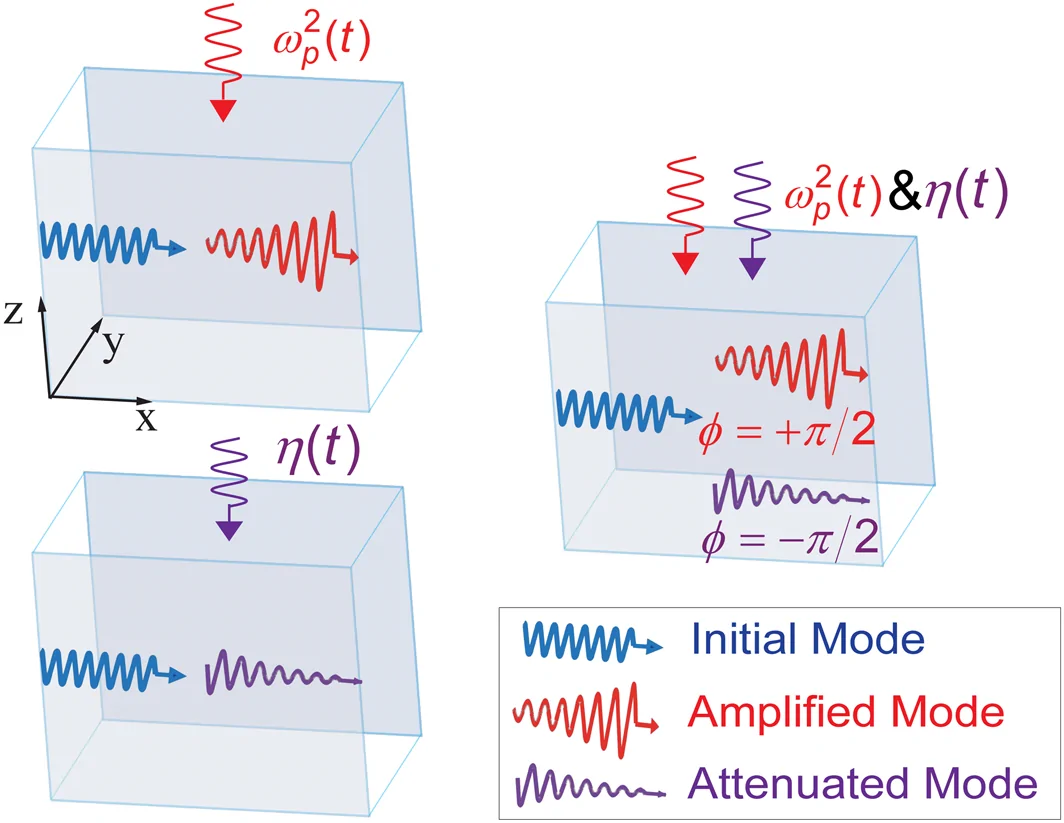

## 1 Introduction

Emerging time‐varying materials have expanded the scope of light–matter interactions by enabling temporal control of material parameters [1, 2, 3, 4, 5, 6, 7, 8, 9]. Photonic time crystals (PTCs), as a distinct class of temporally modulated systems, break time‐translation symmetry while maintaining spatial homogeneity [1, 2, 10, 11]. As a result, electromagnetic energy is no longer conserved within the medium, while momentum conservation remains preserved due to spatial invariance. This unique symmetry configuration enables energy exchange between electromagnetic fields and external modulation sources, leading to a variety of phenomena, including temporal localization analogous to spatial localization [12, 13], control of spontaneous emission [14, 15, 16, 17, 18], optical frequency conversion [19], and nonreciprocal photonic functionalities [20, 21, 22]. The theoretical description of PTCs is commonly established through the Floquet expansion method [14, 17, 23, 24]. In this framework, electromagnetic fields or eigenmodes are decomposed into a series of frequency harmonics separated by integer multiples of the modulation frequency. The coupling among these Floquet harmonics gives rise to an effective eigenvalue equation, from which the system dispersion relation can be derived. This dispersion relation determines light–matter interactions, energy exchange dynamics, and modal stability in temporally modulated medium. Therefore, controlling Floquet coupling and the resulting dispersion characteristics is essential for engineering the position, size, and imaginary‐frequency response of momentum bandgaps in PTCs.

When electromagnetic modes are coupled through temporal Floquet modulation, a bandgap emerges near half of the modulation frequency [1]. Unlike the frequency bandgaps in conventional spatial photonic crystals [25], this bandgap extends along the wavevector axis and is therefore referred to as a momentum bandgap. Within this bandgap, electromagnetic fields continuously exchange energy with the external modulation, leading to exponential amplification or attenuation of the waves. The momentum bandgap is thus a defining feature of PTCs and provides an important platform [1, 2, 26] for exploring nonequilibrium photonic states, parametric amplification, and modulation‐induced energy‐transfer processes.

Previous studies have primarily focused on engineering PTC band structures through periodic modulation of macroscopic effective parameters [23, 27, 28, 29, 30, 31, 32, 33, 34, 35], such as permittivity and permeability. In Drude‐Lorentz dispersive medium, the electromagnetic response is determined by fundamental material parameters, including the plasma frequency [25, 36, 37], resonance frequency [14, 37], and damping factor [30, 38]. Temporal modulation of these parameters provides distinct pathways for tailoring Floquet coupling and band structures. For example, resonance‐frequency modulation exploits slow‐light effects near material resonances to enlarge the momentum bandgap [39]. Plasma‐frequency modulation couples upper and lower polariton branches, forming broadband momentum bandgaps that can be exploited to control spontaneous emission over a wide spectral range [15, 25]. Beyond single‐parameter modulation, Ref. [33] investigated simultaneous temporal modulation of permittivity and permeability, examining the influence of modulation parameters and their relative phase on Floquet responses and reflection–transmission characteristics. More closely related to the present work, Ref. [39] developed a generalized Floquet‐Hamiltonian description of Lorentz‐dispersive PTCs with simultaneous plasma‐frequency and damping modulation, including the effects of their relative phase on the quasifrequency band structure. These studies have established important theoretical foundations for multi‐parameter temporal modulation in dispersive photonic systems.

From a microscopic perspective, Lorentz dispersive medium offers three fundamental modulation channels: the squared plasma frequency $\omega_{p}^{2}$, the squared resonance frequency $\omega_{r}^{2}$, and the damping factor η. The plasma and resonance frequencies primarily determine the strength and spectral characteristics of light–matter coupling, whereas the damping factor governs material loss and associated non‐Hermitian effects. Motivated by their complementary roles, we consider a dual‐channel modulation strategy combining the modulation of $\omega_{p}^{2}$ and η, which respectively control the coupling strength and the loss‐related non‐Hermitian response.

In this work, building on existing multi‐parameter Floquet descriptions, we systematically investigate momentum‐bandgap engineering and phase‐controlled gain‐loss dynamics in a Lorentz‐dispersive PTC through simultaneous modulation of $\omega_{p}^{2}$ and η. Using perturbation theory [2, 40], we obtain analytical expressions for the momentum splitting Δk and complex‐quasifrequency splitting Δω, explicitly relating both quantities to the modulation‐induced coupling coefficients. We show that the interplay between the two channels produces phase‐controlled interference in the modulation‐induced coupling. For appropriate phase relations, the resulting parametric gain compensates for, and can even exceed intrinsic material losses, leading to exponential amplification within the momentum bandgap. In contrast, a different phase relation induces destructive interference, suppressing the modulation‐induced gain and resulting in exponential attenuation. These results establish a general framework for phase‐controlled band‐structure engineering in dispersive PTCs, enabling flexible control of momentum bandgap characteristics and gain‐loss dynamics through dual‐channel modulation of microscopic material parameters.

## 2 Results

### 2.1 The Relationship Between the Electric and Polarization‐Density Fields

To elucidate the underlying physical mechanism, we consider a spatially homogeneous, infinitely extended, nonmagnetic dispersive PTC described by the Lorentz model [24, 41]. In this medium, the electric displacement field D, polarization‐density field P, and electric field E satisfy the relation:

(1) $$\begin{array}{c} D\left(r,t\right)=\varepsilon_{0}E\left(r,t\right)+P\left(r,t\right). \end{array}$$

The material parameters are assumed to possess time‐translational symmetry with a period $T=\frac{2\pi }{\Omega }.$ According to Floquet theory, the eigenfrequencies are periodically replicated with frequency shifts given by integer multiples of the modulation frequency Ω.The frequency interval defined by the first temporal Brillouin zone is [−Ω/2,Ω/2]. Since all Floquet bands can be generated by frequency translations of Ω, the complete band structure can be fully characterized within this zone.

In experimental realizations of time‐varying materials [36, 38, 42], modulation of the plasma frequency is commonly achieved through optical pumping of epsilon‐near‐zero (ENZ) materials, which induces a transient increase in the hot‐electron density N(t). Meanwhile, collisions among hot electrons modify the damping factor. In general, optical pumping process produces an excitation stage associated with an increase in carrier density, followed by a relaxation stage associated with carrier decay, leading to a temporally nonuniform pulse‐like modulation profile [23, 36]. Here, we consider a dual‐channel modulation system to investigate the fundamental effects of plasma‐frequency and damping‐factor modulation on the Floquet band structure. Unlike resonance‐frequency modulation, which directly shifts the material resonance, these two channels primarily modify the effective oscillator strength and dissipation. Their simultaneous modulation provides a convenient setting for investigating interference between modulation‐induced amplification and intrinsic losses. A harmonic modulation model is adopted to isolate the individual contributions of the two modulation channels. The two time‐dependent parameters are described as $\omega_{p}^{2}\left(t\right)=\omega_{p0}^{2}\left[1+m_{p}\;\cos\left(\Omega t\right)\right]$, $\eta \left(t\right)=\eta_{0}\left[1+m_{\eta }\;\cos\left(\Omega t+\phi \right)\right]$, where $m_{p}$ and $m_{\eta }$ denote the modulation depths of the two channels, respectively, and ϕ represents their relative phase. The squared plasma frequency is given by $\omega_{p0}^{2}=\frac{Ne^{2}}{m^{*}\varepsilon_{0}}$, where N denotes the number density of carriers and $m^{*}$ is the effective mass. The parameter $\eta_{0}$ represents the damping factor associated with the material loss.

In this dispersive medium, the polarization‐density field P and electric field E satisfy time‐periodic wave equations and can therefore be expanded using Floquet formalism as $\sum_{n}P_{n}e^{-i\omega_{n}t}$ , $\sum_{n}E_{n}e^{-i\omega_{n}t}$, where $\omega_{n}=\omega_{F}+n\Omega ,$ and $\omega_{F}$ denotes the Floquet eigenfrequency. In the Lorentz model, the polarization‐density field P is governed by the harmonic oscillator equation describing the excitation of bound charges [2]:

(2) $$\begin{array}{c} \frac{\partial^{2}P\left(r,t\right)}{\partial t^{2}}+\eta \left(t\right)\frac{\partial P\left(r,t\right)}{\partial t}+\omega_{r}^{2}P\left(r,t\right)=\varepsilon_{0}\omega_{p}^{2}\left(t\right)E\left(r,t\right). \end{array}$$

The dispersion relation of the dispersive PTC can then be derived from Maxwell's equations and is expressed as $\frac{\omega^{2}\varepsilon \left(\omega ,t\right)}{c^{2}}$, where the material response depends on both the electromagnetic frequency ω and the temporal modulation t. The electric field E and polarization‐density field P components are related through

(3) $$\begin{array}{c} \sum_{m}A_{nm}P_{m}=\varepsilon_{0}\sum_{m}B_{nm}E_{m}, \end{array}$$

where $A_{n,m}=\left(\omega_{r}^{2}-\omega_{n}^{2}\right)\delta_{nm}-i\omega_{m}\eta_{n-m}$, and $B_{n,m}=\omega_{p,n-m}^{2}$. The Floquet expansion is truncated to n=−N,…,N. Here, $\omega_{r}$ denotes the resonance frequency. The resulting polarization‐density field can be expressed as $P=\varepsilon_{0}\chi E$, where $\chi =A^{-1}B$. This relation establishes the connection between the electric field and polarization‐density field and provides the foundation for deriving the dispersion relation of the dispersive PTC.

### 2.2 Band Structure Calculation of PTCs

Combining Maxwell's equations [24]:

(4) $$\begin{array}{c} \nabla \times E=-\mu_{0}\frac{\partial H}{\partial t}, \end{array}$$

(5) $$\begin{array}{c} \nabla \times H=\frac{\partial D}{\partial t}=\varepsilon_{0}\frac{\partial E}{\partial t}+\frac{\partial P}{\partial t}, \end{array}$$

with the electromagnetic wave equation $\nabla \times \nabla \times E=-\frac{1}{c^{2}}\frac{\partial^{2}E}{\partial t^{2}}-\frac{1}{\varepsilon_{0}c^{2}}\frac{\partial^{2}P}{\partial t^{2}}$, the eigenvalue equation of the dispersive PTC can be written as

(6) $$\begin{array}{c} \sum_{m}\left[\left(\frac{\omega_{n}^{2}}{c^{2}}-k^{2}\right)\delta_{nm}+\frac{\omega_{n}^{2}}{c^{2}}\chi_{nm}\right]E_{m}=0, \end{array}$$

where $\chi_{nm}={\left[A^{-1}B\right]}_{nm}.$ For each prescribed wavevector k, the corresponding Floquet eigenfrequency $\omega_{F}$ can be obtained by solving Equation (6). The collection of these eigenfrequencies forms the band structure of the dispersive PTC, as shown in Figure 1a–c. The temporal modulation lifts the degeneracy between the coupled Floquet branches, giving rise to a momentum bandgap, as illustrated in Figure 1c. The two characteristic wavevectors $k_{\pm }$ define the momentum‐gap width. Meanwhile, the complex quasifrequencies $\omega_{\pm }$ characterize the imaginary‐frequency separation. For a given wavevector k, the different frequency components $\omega_{n}=\omega_{F}+n\Omega$ collectively constitute a Floquet eigenmode. Accordingly, the electric field E can be expressed as $E\left(r,t\right)=\sum_{n=-\infty }^{+\infty }E_{n}\left(\omega_{F}\right)\exp\left(-i\omega_{n}t\right)\exp\left(ik\cdot r\right).$ The corresponding amplitudes of the Floquet harmonics are shown in Figure 1d. The contribution of the *n*th Floquet harmonic is characterized by the normalized electric‐field amplitude $W_{n}={\left|E_{n}\right|}^{2}/\sum {\left|E_{m}\right|}^{2}$. The zeroth‐ and minus‐first‐order harmonics dominate the Floquet eigenmode, whereas higher‐order harmonics contribute only weakly and can therefore be treated as perturbative corrections. Under this approximation, the Floquet eigenvalue equation can be reduced to a two‐mode model involving only the n=0 and n=−1 harmonics:

(7) $$\begin{array}{c} \left[\begin{array}{cc} D_{-1} & M_{-1,0}\\ M_{0,-1} & D_{0} \end{array}\right]\left[\begin{array}{c} E_{-1}\\ E_{0} \end{array}\right]=\left[\begin{array}{c} 0\\ 0 \end{array}\right], \end{array}$$

where $D_{n}=\frac{\omega_{n}^{2}}{c^{2}}-k^{2}+\frac{\omega_{n}^{2}}{c^{2}}\chi_{nn},n=0,-1$ describes the dispersive response of the nth Floquet harmonic, while $M_{nm}=\frac{\omega_{n}^{2}}{c^{2}}\chi_{nm},n\neq m$ represents the coupling strength between the nth and mth Floquet harmonics. For coupling between positive‐ and negative‐frequency replicas of the same polariton branch, the crossing condition is generally satisfied near Ω/2. Because the two uncoupled Floquet replicas have opposite slopes at the crossing point, the center of the resulting momentum bandgap is approximately flat. The Floquet modes within this bandgap generally acquire complex eigenfrequencies, corresponding to temporal amplification or attenuation. Net amplification occurs when the modulation‐induced parametric gain exceeds the intrinsic material loss.

**FIGURE 1:**
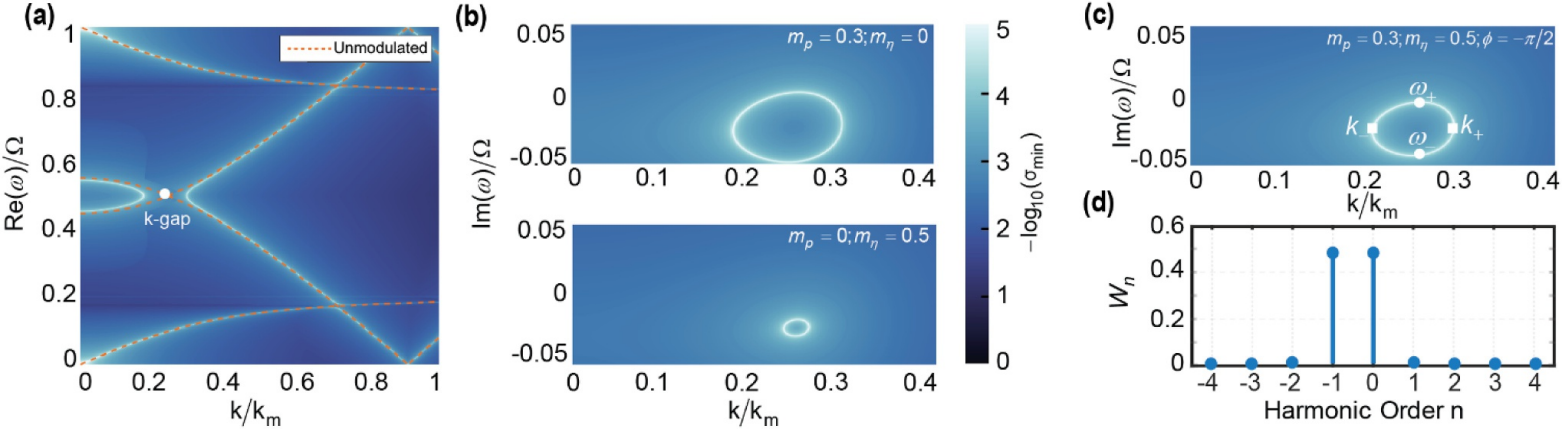
Momentum bandgap formation in a dispersive PTC. Parameter setting: $\omega_{p0}=0.4\;\Omega ,\omega_{r}=0.2\;\Omega ,m_{p}=0.3,\eta_{0}=0.06\;\Omega ,f_{m}=0.6\text{GHz}\;$. (a) Real eigenfrequency band structure. The white marker denotes the crossing point, where temporal modulation opens a momentum bandgap along the wavevector direction. The orange dashed lines represent the band dispersion curves of the unmodulated system. (b) Imaginary part of the Floquet eigenfrequency dispersion for plasma‐frequency modulation only (upper panel) and damping modulation only (lower panel) as a function of normalized wavevector $k/k_{m}$ near the bandgap. (c) Imaginary quasifrequency dispersion under dual‐channel modulation at a relative phase of ϕ=−π/2. (d) Normalized amplitudes of the Floquet harmonic components at the selected bandgap point. The relative contribution of the *n*th Floquet harmonic is quantified by the normalized electric‐field amplitude $W_{n}={\left|E_{n}\right|}^{2}/\sum {\left|E_{m}\right|}^{2}$. The dominant contributions come from the n=0 and n=−1 harmonics, confirming that the bandgap is mainly formed by two‐mode Floquet coupling.

In this work, we focus on the intrabranch momentum bandgap near Ω/2. The opposite dispersion slopes of these modes provide a relatively simple and symmetric coupling configuration, allowing us to isolate the effects of dual‐channel interference on momentum‐bandgap splitting and gain–loss characteristics. In addition to intrabranch coupling, interbranch coupling between different polariton branches can occur at distinct crossing points. Specifically, the positive‐frequency upper polariton branch can couple to a Floquet‐shifted negative‐frequency lower polariton branch. Since the two interacting Floquet branches generally possess different group velocities, the resulting hybrid momentum bandgap exhibits a finite dispersion slope. Moreover, the modes within this hybrid bandgap retain nonzero group velocities and can therefore propagate while simultaneously experiencing temporal amplification or attenuation.

### 2.3 Influence of Dual‐Channel Modulation on the Momentum Bandgap

To quantify the effect of dual‐channel modulation on the momentum bandgap, we employ perturbation theory [40] to derive analytical expressions for its size and position. We focus on the same‐branch momentum bandgap near $\omega_{F}=\Omega /2$. The system is assumed to operate in the weak‐modulation regime $m_{p}\ll 1,m_{\eta }\ll 1.$ Since the bandgap is dominated by the degeneracy between n=0 and n=−1 Floquet harmonics, the eigenvalue equation reduces to $D_{-1}D_{0}=0.$ Here, $D_{n}$ (n=0,−1) in Equation (7) retains the dispersion relation of the dispersive medium. Although neighboring dispersion branches are folded by integer multiples of the modulation frequency Ω, no off‐diagonal coupling exists in the absence of temporal modulation. Consequently, the folded branches intersect without hybridization. We denote the crossing point as $\left(k_{c},{\tilde{\omega }}_{c}\right)$. The material loss shifts this point downward in the complex‐frequency plane, corresponding to a negative imaginary‐frequency offset of the momentum bandgap. For the temporal convention exp(−iωt), the positive decay rate $\gamma_{0}$ can be estimated from $\gamma_{0}=\frac{\text{Im}D_{0}}{\partial_{\omega }\text{Re}D_{0}}.$

Accordingly, the complex frequency at the unperturbed degeneracy can be written as ${\tilde{\omega }}_{c}=\omega_{c}-i\gamma_{0}.$ The off‐diagonal matrix elements $M_{nm}$ describe the effective coupling between Floquet harmonics induced by temporal modulation. Near the crossing point, the two‐mode eigenvalue equation becomes $D_{-1}D_{0}-M_{0,-1}M_{-1,0}=0.$ To analyze the bandgap formation around the degeneracy, we introduce small perturbations $\omega ={\tilde{\omega }}_{c}+\delta \omega .$ After incorporating the intrinsic material loss into the complex crossing frequency ${\tilde{\omega }}_{c}$, the diagonal terms can be expanded to first order as $D_{n}\left(k,{\tilde{\omega }}_{c}+\delta \omega \right)=\alpha_{n}\delta \omega +k_{c}^{2}-k^{2},n=0,-1$, where the frequency derivative coefficient $\alpha_{n}=\frac{2\omega_{n}}{c^{2}}+\frac{2\omega_{n}}{c^{2}}\chi_{nn}+\frac{\omega_{n}^{2}}{c^{2}}{\frac{\partial \chi_{n,n}}{\partial \omega }|}_{c}$. Here, the subscript *c* indicates evaluation at the unmodulated complex‐frequency crossing point. Substituting these expressions into Equation (7) gives

(8) $$\begin{array}{c} \alpha_{0}\alpha_{-1}{\left(\delta \omega \right)}^{2}+\left(k_{c}^{2}-k^{2}\right)\left(\alpha_{0}+\alpha_{-1}\right)\delta \omega +{\left(k_{c}^{2}-k^{2}\right)}^{2}=M_{0,-1}M_{-1,0}, \end{array}$$

which provides the local relationship between frequency and wavevector splitting. At the central wavevector, where $k=k_{c}$, the two complex eigenfrequencies are therefore $\omega_{\pm }={\tilde{\omega }}_{c}\pm \sqrt{\frac{M_{0,-1}M_{-1,0}}{\alpha_{0}\alpha_{-1}}}.$ Similarly, at the central frequency, where δω=0, the two positive‐real‐wavevector solutions can be written as $k_{\pm }=\sqrt{k_{c}^{2}\mp \sqrt{M_{0,-1}M_{-1,0}}}.$ The solutions $\omega_{\pm }$ and $k_{\pm }$ characterize the four local edge points of the modulation‐induced momentum band structure(see Supporting Information S1: Section S1). Therefore, the momentum bandgap size along the wavevector and frequency directions are given by $\Delta \omega =2\sqrt{\frac{M_{0,-1}M_{-1,0}}{\alpha_{0}\alpha_{-1}}}$, $\Delta k=\sqrt{k_{c}^{2}+\sqrt{M_{0,-1}M_{-1,0}}}-\sqrt{k_{c}^{2}-\sqrt{M_{0,-1}M_{-1,0}}}$.

Figure 2a compares the effects of the two individual modulation channels. Both plasma‐frequency modulation and damping‐factor modulation generate momentum bandgap, and in the weak‐modulation regime the bandgap size increases nearly linearly with the modulation depth. This behavior indicates that the effective Floquet coupling induced by each channel is proportional to the corresponding modulation amplitude. However, the two channels exhibit different modulation efficiencies due to their distinct physical origins. Plasma‐frequency modulation mainly modifies the coupling between the electromagnetic field and the polarization‐density response, whereas damping‐factor modulation introduces a tunable non‐Hermitian coupling pathway. These distinct contributions provide the basis for understanding the interference effects arising from simultaneous modulation.

**FIGURE 2:**
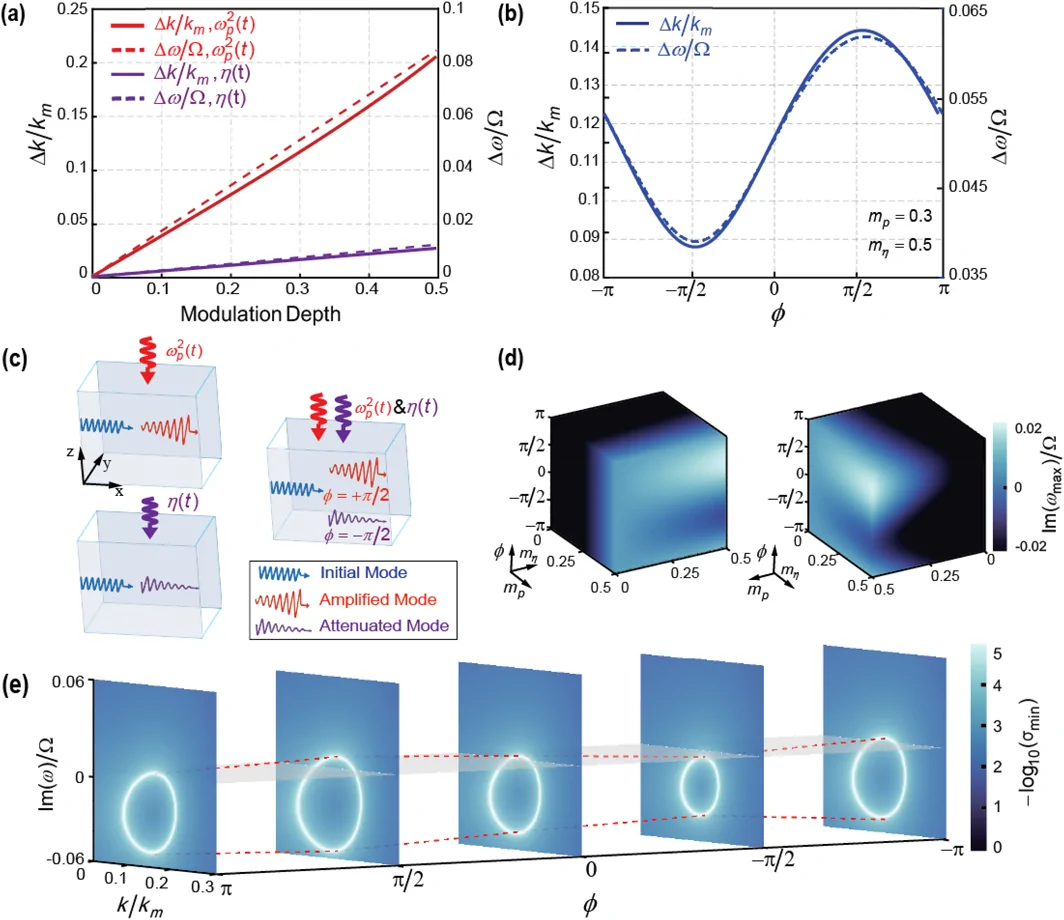
Dual‐channel modulation and phase‐control of the momentum bandgap. Parameter setting: $\omega_{p0}=0.4\;\Omega ,\omega_{r}=0.2\;\Omega ,\eta_{0}=0.06\;\Omega ,f_{m}=0.6\;\text{GHz}.$ (a) Normalized bandgap splitting induced by single‐channel modulation. (b) Phase‐dependent variation of the bandgap splitting for fixed modulation depths $m_{p}=0.3$ and $m_{\eta }=0.5$, revealing that the relative phase ϕ provides an additional degree of freedom for controlling bandgap splitting. (c) Schematic illustration of a dispersive PTC with modulations of $\omega_{p}^{2}\left(t\right)$ and η(t), showing the emergence of temporal amplification or attenuation for different modulation schemes. (d) Three‐dimensional parameter‐space distribution of $\text{Im}(\omega_{\max}$). Here, $\text{Im}(\omega_{\max}$) represents the maximum imaginary component of the Floquet eigenfrequencies within the bandgap. The two plots present different viewing angles to illustrate its dependence on the three modulation parameters. (e) Phase‐dependent evolution of the imaginary‐frequency distribution near the bandgap, showing that the relative phase modifies the gain‐loss characteristics of the bandgap modes. The red dashed lines trace the two Floquet quasifrequency branches, $\omega_{+}$ and $\omega_{-}$, respectively. The gray plane denotes Im(ω)=0.

To investigate the combined effect of the two modulation channels, we analyze the off‐diagonal susceptibility. The coupling matrix elements are defined as $M_{nm},n\neq m.$ Under the weak‐modulation condition, the off‐diagonal susceptibility can be expressed as

(9) $$\begin{array}{c} \chi_{nm}≃\frac{\omega_{p,n-m}^{2}}{a_{n}}+\frac{i\omega_{m}\eta_{n-m}\omega_{p0}^{2}}{a_{n}a_{m}},n\neq m, \end{array}$$

where $a_{n}=\omega_{r}^{2}-\omega_{n}^{2}-i\omega_{n}\eta_{0}$ (see Supporting Information S1: Section S2). The first term in Equation (9) corresponds to the coupling induced by plasma‐frequency modulation, whereas the second term arises from the temporal modulation of the damping factor. The former modifies the oscillator strength of the Lorentz response, while the latter introduces a non‐Hermitian coupling channel through the time‐dependent modulation of material dissipative response. For convenience, the two coupling contributions are expressed as $M_{-1,0}=A_{1}+B_{1}e^{i\phi },M_{0,-1}=A_{2}+B_{2}e^{-i\phi },$ where $A_{1}$ and $A_{2}$ denote the contributions from plasma‐frequency modulation, while $B_{1}$ and $B_{2}$ denote the contributions from damping‐factor modulation, ϕ denote their relative phase. The coefficients $A_{1}$ and $A_{2}$ are proportional to the plasma‐frequency modulation depth $m_{p}$, whereas $B_{1}$ and $B_{2}$ are proportional to the damping‐factor modulation depth $m_{\eta }$. For pure plasma‐frequency modulation, the coupling product reduces to $M_{-1,0}M_{0,-1}=A_{1}A_{2},$ which is independent of the relative phase. Likewise, for pure damping‐factor modulation, $M_{-1,0}M_{0,-1}=B_{1}B_{2}$ is obtained. In both single‐channel cases, the relative phase associated with the forward and backward Floquet coupling cancel each other in the coupling product, and therefore the bandgap size is determined solely by the modulation amplitude. For simultaneous modulation, the coupling product becomes

(10) $$\begin{array}{c} M_{-1,0}M_{0,-1}=A_{1}A_{2}+B_{1}B_{2}+A_{1}B_{2}e^{-i\phi }+A_{2}B_{1}e^{i\phi }, \end{array}$$

where the phase‐dependent cross terms $A_{1}B_{2}e^{-i\phi }$ and $A_{2}B_{1}e^{i\phi }$ describe the interference between the plasma‐frequency modulation and damping‐factor modulation. Consequently, both modulation amplitudes $m_{p}$ and $m_{\eta }$, and their relative phase ϕ, affect the size and complex phase of the effective Floquet coupling. Constructive interference enhances the effective coupling and enlarges the momentum bandgap, as shown in Figure 2b. Conversely, destructive interference suppresses the coupling strength and reduces the bandgap size. Because damping‐factor modulation introduces a non‐Hermitian contribution to the coupling, the relative phase also modifies the imaginary part of the band structure, and therefore controls the amplification and attenuation of Floquet modes.

### 2.4 Phase‐Controlled Amplification and Attenuation in Dual‐Channel‐Modulated PTCs

We next analyze how the relative phase in dual‐channel modulation controls the amplification and attenuation characteristics of Floquet modes. As shown in Equation (10), the relative phase modifies the effective coupling through the phase‐dependent cross terms $A_{1}B_{2}e^{-i\phi }+A_{2}B_{1}e^{i\phi }$. Near the degeneracy point, the two momentum bandgap branches projected onto the complex‐frequency plane can therefore be written as $\omega_{\pm }=\omega_{c}-i\gamma_{0}\pm \delta \omega \left(\phi \right),$ where $\gamma_{0}>0$ represents the frequency shift caused by the intrinsic material loss. At the center of the momentum bandgap, the modulation‐induced frequency splitting occurs along the imaginary‐frequency axis. It counteracts the negative imaginary‐frequency offset caused by the intrinsic material loss. The imaginary parts of the two branches can therefore be expressed as $\text{Im}\left[\omega_{\pm }\right]=-\gamma_{0}\pm \left|\text{Im}\left[\delta \omega \left(\phi \right)\right]\right|.$ Exponential amplification occurs when the upper branch satisfies $-\gamma_{0}+\left|\text{Im}\left[\delta \omega \left(\phi \right)\right]\right|>0$, whereas both branches remain attenuating when he upper branch satisfies $-\gamma_{0}+\left|\text{Im}\left[\delta \omega \left(\phi \right)\right]\right|<0$. Figure 2c illustrates the physical mechanism associated with different modulation schemes. Under plasma‐frequency modulation alone, the Floquet coupling can generate a mode with net amplification. For the parameters considered here, the Floquet coupling induced by damping modulation alone is insufficient to overcome the intrinsic material loss. When both modulation channels are simultaneously applied, the system response is determined not only by the modulation amplitudes, but also by their relative phase. The two modulation pathways interfere constructively or destructively, thereby enhancing or suppressing the effective Floquet coupling. Consequently, the Floquet response can be continuously tuned between amplification and attenuation by adjusting the modulation amplitudes and relative phase.

Figure 2d presents the variation of $\text{Im}(\omega_{\max}$) in the three‐dimensional parameter space of $m_{p}$, $m_{\eta }$, and ϕ, with the damping‐induced frequency shift taken into account. Here, $\text{Im}(\omega_{\max}$) denotes the maximum imaginary part of the complex frequency within the momentum bandgap and is used to distinguish whether the system response is amplification‐ or attenuation‐dominated. The results indicate that $\text{Im}(\omega_{\max}$) is governed by the combined effect of the two modulation amplitudes and their relative phase, rather than by any individual modulation parameter. For appropriate parameter combinations, $\text{Im}(\omega_{\max}$) becomes positive, indicating that the modulation‐induced parametric gain overcomes the intrinsic material loss. Otherwise, $\text{Im}(\omega_{\max}$) remains negative, corresponding to attenuation‐dominated response. Figure 2e further shows the imaginary‐frequency distributions near the momentum bandgap for ϕ. The results directly illustrate how relative phase ϕ shifts the momentum bandgap branches in the complex‐frequency plane. By modifying the phase dependent Floquet coupling, the relative phase ϕ continuously tunes the imaginary part of the band structure and thereby controls the transition between amplification‐ and attenuation‐dominated regimes. From an experimental perspective, optical pumping can induce variations in both the effective plasma frequency and damping factor, as reported in studies of ultrafast time‐varying materials, including ENZ material [36, 38, 43]. Alternatively, electronically tunable metamaterials or transmission‐line platforms may provide separate control of the effective oscillator strength and dissipation through synchronized external driving. These approaches offer potential routes toward dual‐parameter modulation, although independent phase control and the assumed modulation depths and frequencies remain experimentally challenging.

### 2.5 Time‐Domain Verification of Phase‐Controlled Amplification and Attenuation

To validate the phase‐controlled mechanism and its influence on Floquet wave dynamics, we perform time‐domain simulations of electromagnetic‐wave propagation in the dispersive PTC. The simulations are based on a one‐dimensional model implemented using the coupled FDTD‐ADE method [44] for a time‐varying Lorentz dispersive Medium. The electromagnetic wave propagates along the *x*‐direction, and the spatiotemporal evolution of the electric‐field component $E_{y}$ is recorded. Perfectly matched layers are applied at both boundaries to suppress reflections. The squared plasma frequency ${\;\omega }_{p}^{2}\left(t\right)$ and damping factor η(t) are periodically modulated with the same modulation frequency, while their modulation depth and relative phase can be independently controlled. This dual‐channel modulation simultaneously modifies the dispersive response and dissipative properties of the medium, thereby influencing the energy exchange between the electromagnetic field and the time‐varying material.

As shown in Figure 3a, a smooth temporal switching function is introduced to control the modulation process. The orange dashed lines mark the onset (t=10ns) and completion (t=170ns) of the modulation process, whereas the red dashed lines indicate the times at which the modulation becomes fully activated (t=20 ns) and begins to be switched off (t=160ns). Such a smooth switching profile suppresses spurious transient responses induced by abrupt temporal discontinuities and enables a clear observation of the wave‐packet evolution after the modulation is removed.

**FIGURE 3:**
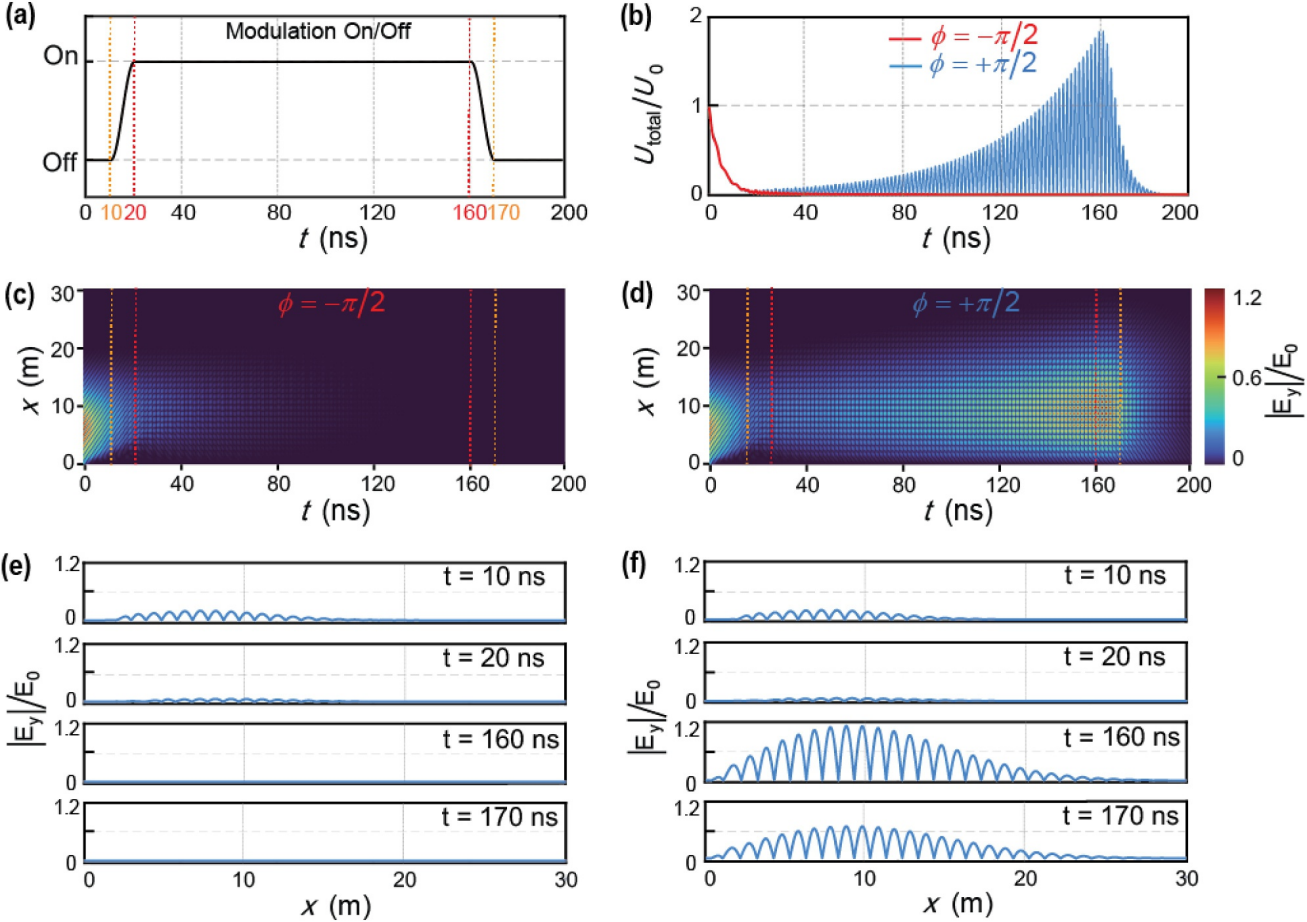
Time‐domain simulations of phase‐controlled amplification and attenuation. Parameter setting: $\omega_{p0}=0.4\;\Omega ,\omega_{r}=0.2\;\Omega ,m_{p}=0.3,m_{\eta }=0.5,\eta_{0}=0.06\;\Omega ,f_{m}=0.6\;\text{GHz}$. (a) A smooth temporal switching function to control the modulation process. The orange dashed lines mark the onset t=10ns and completion t=170ns of the modulation process, whereas the red dashed lines indicate the times at which the modulation becomes fully activated t=20 ns and begins to be switched off t=160ns. (b) The normalized integrated electric‐field intensity $U_{\text{total}}/U_{0}$ for ϕ=+π/2 and ϕ=−π/2. (c), (d) Spatiotemporal distributions of the normalized electric field for ϕ=−π/2 and ϕ=+π/2. The frequency of incident wave is $f_{\text{inc}}=0.3\;\text{GH}z$, the carrier cycles is $N_{\text{inc}}=12$. (e), (f) Electric‐field snapshots $E_{y}$ at selected times for ϕ=−π/2 and ϕ=+π/2, showing the wave‐packet evolution before, during, and after the modulation.

The incident excitation is a Gaussian‐modulated wave packet with a central frequency selected near Ω/2. A sufficiently large number of carrier cycles is used to reduce the spectral bandwidth, ensuring that the excitation mainly overlaps with the targeted Floquet‐coupling region. At this frequency, the momentum bandgap and the associated amplification or attenuation effects are most pronounced. Figure 3b compares the normalized integrated electric‐field intensity $U_{\text{total}}\left(t\right)$ for two opposite relative phases, where $U_{\text{total}}\left(t\right)=\sum_{i}{\left|E_{y}\left(x_{i},t_{n}\right)\right|}^{2}\Delta x$. The reference electric‐field intensity $U_{0}$ is defined as the maximum value of $U_{\text{total}}\left(t\right)$ within the initial unmodulated time interval. For one phase configuration, ϕ=−π/2, $U_{\text{total}}$ decreases rapidly after the modulation is activated and remains suppressed, indicating a Floquet attenuation regime. In contrast, for the opposite phase configuration, ϕ=π/2, $U_{\text{total}}$ increases continuously during the modulation interval, demonstrating net amplification. This comparison confirms that the system response can be switched between amplification and attenuation solely by tuning the relative phase while keeping all other parameters unchanged.

Figure 3c,d present the corresponding spatiotemporal electric field distributions, while Figure 3e,f display the snapshots of electric‐field component $E_{y}$ captured at distinct modulations. These results clearly reveal that the propagation behaviors in the spatiotemporal domain exhibit a pronounced divergence under different phase configurations. For the case of ϕ=−π/2, the spatiotemporal electric field remains localized near the incident region during the gradual activation of the modulation, before the modulation reaches its full strength. Once the modulation is fully activated, the spatiotemporal electric field decays progressively, which corresponds to an attenuation‐dominated response (Figure 3c). In contrast, for the case of ϕ=π/2, the spatiotemporal electric field keep increasing continuously after the modulation is fully activated, indicating an amplification‐dominated response (Figure 3d). These findings further verify that the relative phase in the dual‐channel modulation governs both the direction and efficiency of energy exchange between the electromagnetic wave and the temporally modulated medium. It is worth noting that at t=10ns, when the modulation is still in the gradual activation phase, the evolution of electric field under the two different phase configurations is identical. Moreover, at t=20ns, the system has just reached the designed modulation strength, so the modulation‐induced amplification or attenuation remains weak, leading to highly similar electric field profile evolutions at these early stages. As the modulation time reaches t=160ns, the electric field for ϕ=−π/2 case undergoes strong attenuation, while the field for the ϕ=π/2 case is significantly amplified. After the modulation is switched off at t=170ns, both the amplification and attenuation process terminate, and the resulting wave packets continue to propagate in the restored unmodulated medium. These snapshots provide spatially resolved validation for the phase‐controlled amplification and attenuation phenomena observed in the spatiotemporal distributions.

## 3 Conclusion

In conclusion, this work addresses the problem of limited band control in dispersive PTCs under single‐parameter modulation. To overcome this limitation, we propose a dual‐channel modulation scheme in which the squared plasma frequency and damping factor are simultaneously modulated in time. Using perturbation theory, we analyze how these two microscopic modulation channels affect the momentum bandgap and the associated complex‐frequency response. For individual modulation channels, we show that modulation of either the squared plasma frequency or the damping factor independently modifies the momentum bandgap, with the bandgap size increasing nearly linearly with the corresponding modulation depth in the weak‐modulation regime. By incorporating the intrinsic material loss through the complex‐frequency shift, we further reveal the competition between modulation‐induced parametric gain and dissipative loss. When both modulation channels are simultaneously applied, the momentum bandgap response is no longer determined by the modulation amplitudes alone but is governed by their mutual interference. The modulation amplitudes and relative phase jointly determine the effective Floquet coupling and consequently control the bandgap characteristics and complex‐frequency response. In particular, the relative phase enables constructive or destructive interference between the two modulation pathways, providing continuous control over the transition between attenuation‐ and amplification‐dominated regimes. These results establish a general analytical framework for phase‐controlled band engineering in dispersive PTCs and demonstrate the potential of multi‐parameter temporal modulation for controlling non‐Hermitian photonic dynamics.

## Author Contributions

**Hongzhi Chen:** methodology, software, visualization, writing – original draft, investigation, validation. **Keyu Jin:** methodology, investigation, validation, software. **Yingjie Wu:** supervision, writing – review and editing, methodology. **Bingtao Gao:** methodology, supervision, writing – review and editing. **Hongsheng Chen:** methodology, funding acquisition, writing – review and editing, supervision, conceptualization, project administration. **Lian Shen:** conceptualization, funding acquisition, methodology, writing – review and editing, supervision, project administration.

## Conflicts of Interest

The authors declare no conflicts of interest.

## Supporting information


Supporting Information S1

## Data Availability

All relevant data presented in the paper are available upon reasonable request.
