# Supplementary material for: Phase‐Controlled Momentum Bandgaps in Dual‐Channel Dispersive Photonic Time Crystals
Source: Nanophotonics. 2026 Nov 13;15(22):e70344. doi: 10.1002/nap2.70344 (PMC13764568; doi:10.1002/nap2.70344)
Supplement: Supplementary file 1 [file NAP2-15-e70344-s001.docx]

**Supplementary Material for**

**Phase-Controlled Momentum Bandgaps in Dual-Channel Dispersive Photonic Time Crystals**

Hongzhi Chen^1,2,3,#^, Keyu Jin^1,2,3,#^, Yingjie Wu^1,2,3^, Bingtao Gao^1,2,3^, Hongsheng Chen^1,2,3,*^, and Lian Shen^1,2,3,*^

*^1^State Key Laboratory of Extreme Photonics and Instrumentation, ZJU-Hangzhou Global Scientific and Technological Innovation Center, Zhejiang University, Hangzhou, China.*

*^2^The Electromagnetics Academy at Zhejiang University, College of Information Science and Electronic Engineering, Zhejiang University, Hangzhou, China.*

*^3^Zhejiang Key Lab. of Intelligent Electromagnetic Control and Advanced Electronic Integration, Jinhua Institute of Zhejiang University, Zhejiang University, Jinhua, China.*

^#^These authors contributed equally to this work: Hongzhi Chen, Keyu Jin.

^*^Correspondence to: [hansomchen@zju.edu.cn](mailto:hansomchen@zju.edu.cn), [lianshen@zju.edu.cn](mailto:lianshen@zju.edu.cn).

**1. Momentum bandgap size**

**2. Susceptibility matrix under weak modulation approximation**

**3. FDTD-ADE initial and boundary conditions**

**4. Analysis of single-channel modulation and initial excitation phase**

**5. Phase-dependent field evolution under nondegenerate excitation**

**1. Momentum bandgap size**

Within the two-mode Floquet approximation, the diagonal terms corresponding to the $n=0$ and $n=-1$ harmonics are given by $D_{n}=\frac{\omega_{n}^{2}}{c^{2}}-k^{2}+\frac{\omega_{n}^{2}}{c^{2}}\chi_{nn}, n=0,-1$, where the diagonal susceptibility is $\chi_{nn}=\frac{\omega_{p0}^{2}}{a_{n}}$, $\omega_{n}=\tilde{\omega}_{c}+n\Omega$. To retain the exact quadratic wavevector dependence, we introduce $F_{n}=\frac{\omega_{n}^{2}}{c^{2}}\left( 1+\chi_{n,n} \right)$, the diagonal term can be expressed as $D_{n}=F_{n}-k^{2}$.

Let $(k_{c},\tilde{\omega}_{c})$ denote the complex-frequency crossing point of the two unmodulated Floquet branches, satisfying $D_{n}(k_{c},\tilde{\omega}_{c})=0$. Accordingly, $F_{n}\left( \tilde{\omega}_{c} \right)=k_{c}^{2}$.

$$\begin{aligned} F_{n}\left( \tilde{\omega}_{c}+\delta\omega\right)=k_{c}^{2}+\left. \frac{\partial F_{n}}{\partial\omega} \right|_{c}\delta\omega+O[\left( \delta\omega\right)^{2}], \#\left( S1 \right) \end{aligned}$$

$$\begin{aligned} \left. \frac{\partial F_{n}}{\partial\omega} \right|_{c}=\frac{2\omega_{n}}{c^{2}}+\frac{2\omega_{n}}{c^{2}}\chi_{nn}+\frac{\omega_{n}^{2}}{c^{2}}\left. \frac{\partial\chi_{n,n}}{\partial\omega} \right|_{c}=\frac{1}{c^{2}}\left[ 2\omega_{n}\left( 1+\frac{\omega_{p0}^{2}}{a_{n}} \right)+\frac{\omega_{n}^{2}\omega_{p0}^{2}\left( 2\omega_{n}+i\eta_{0} \right)}{a_{n}^{2}} \right],\#\left( S2 \right) \end{aligned}$$

where the frequency derivative coefficient is $\alpha_{n}=\left. \frac{\partial F_{n}}{\partial\omega} \right|_{c}$. Here, the subscript c indicates evaluation at the unmodulated complex-frequency crossing point. The intrinsic damping coefficient $\eta_{0}$ is fully retained, without invoking a weak-loss approximation.

The diagonal dispersion terms can be approximated as

$$\begin{aligned} D_{n}\left( k,\tilde{\omega}_{c}+\delta\omega\right)=\alpha_{n}\delta\omega+k_{c}^{2}-k^{2}.\#(S3) \end{aligned}$$

Substituting Eq. (S3) into the two-mode dispersion equation $D_{-1}D_{0}-M_{0,-1}M_{-1,0}=0.$ where ($M_{0,-1})$ and $(M_{-1,0})$ denote the off-diagonal coupling matrix elements between the two retained Floquet harmonics, the resulting dispersion relation becomes

$$\begin{aligned} \alpha_{0}\alpha_{-1}\left( \delta\omega\right)^{2}+\left( k_{c}^{2}-k^{2} \right)\left( \alpha_{0}+\alpha_{-1} \right)\delta\omega+\left( k_{c}^{2}-k^{2} \right)^{2}=M_{0,-1}M_{-1,0}. \#\left( S4 \right) \end{aligned}$$

Equation (S4) describes the local frequency-wavevector relationship while retaining the intrinsic Lorentz dispersion, finite material loss, and exact quadratic wavevector dependence.

**(1) Frequency splitting at the central wavevector**

At the central wavevector ($k=k_{c}$), Eq. (S4) reduces to ${(\delta\omega)}^{2}=\frac{M_{0,-1}M_{-1,0}}{\alpha_{0}\alpha_{-1}}$, the two complex eigenfrequencies are therefore $\omega_{\pm}=\tilde{\omega}_{c}\pm\sqrt{\frac{M_{0,-1}M_{-1,0}}{\alpha_{0}\alpha_{-1}}}$. Consequently, complex-frequency splitting at the crossing point is given by $\Delta\omega=2\sqrt{\frac{M_{0,-1}M_{-1,0}}{\alpha_{0}\alpha_{-1}}}$.

**(2) Wavevector splitting at the central complex frequency**

At the central complex frequency ($\delta\omega=0$), Eq. (S4) becomes ${(k_{c}^{2}-k^{2})}^{2}=M_{0,-1}M_{-1,0}$, the two positive-real-wavevector solutions can be written as $k_{\pm}=\sqrt{k_{c}^{2}\mp\sqrt{M_{0,-1}M_{-1,0}}}$. Accordingly, the full wavevector separation at the central complex frequency is $\Delta k=\sqrt{k_{c}^{2}+\sqrt{M_{0,-1}M_{-1,0}}}-\sqrt{k_{c}^{2}-\sqrt{M_{0,-1}M_{-1,0}}}$.

**2. Susceptibility matrix under weak modulation approximation**

Starting from Eq. (3): $\sum_{m} A_{nm}P_{m}=\varepsilon_{0}\sum_{m} B_{nm}E_{m},$ where $A_{n,m}=(\omega_{r}^{2}-\omega_{n}^{2})\delta_{nm}-i\omega_{m}\eta_{n-m}$, and $B_{n,m}=\omega_{p,n-m}^{2}$. The resulting polarization-density field can be expressed as $\boldsymbol{P}=\varepsilon_{0}\boldsymbol{\chi E}$, where $\boldsymbol{\chi}\boldsymbol{=}\boldsymbol{A}^{-1}\boldsymbol{B}$. We separate the matrices into their static and modulation-induced components:

$\begin{aligned} \boldsymbol{A}=\boldsymbol{A}_{0}+\delta\boldsymbol{A},\#\left( S5 \right) \end{aligned}$

$$\begin{aligned} \boldsymbol{B}=\boldsymbol{B}_{0}+\delta\boldsymbol{B},\#\left( S6 \right) \end{aligned}$$

where ${(A_{0})}_{nm}=\left( \omega_{r}^{2}-\omega_{n}^{2}-i\omega_{n}\eta_{0} \right)\delta_{nm}$, ${(\delta A)}_{nm}=-i\omega_{m}\eta_{n-m},n\neq m$, ${(B_{0})}_{nm}=\omega_{p0}^{2}\delta_{nm}$,${(\delta B)}_{nm}=\omega_{p,n-m}^{2}$.

$$\begin{aligned} \boldsymbol{A}^{-1}=\left( \boldsymbol{A}_{0}+\delta\boldsymbol{A} \right)^{-1}=\left( \boldsymbol{I}+\boldsymbol{A}_{0}^{-1}\delta\boldsymbol{A} \right)^{-1}\boldsymbol{A}_{0}^{-1}\simeq\boldsymbol{A}_{0}^{-1}-\boldsymbol{A}_{0}^{-1}\delta\boldsymbol{A}\boldsymbol{A}_{0}^{-1},\#\left( S7 \right) \end{aligned}$$

where ${{(A}_{0}^{-1})}_{nm}=diag(\frac{1}{a_{n}})\delta_{nm}$. Terms of second and higher orders in the modulation depths are neglected. So $\boldsymbol{\chi}\simeq\boldsymbol{A}_{0}^{-1}\boldsymbol{B}_{0}+\boldsymbol{A}_{0}^{-1}\delta\boldsymbol{B}-\boldsymbol{A}_{0}^{-1}\left( \delta\boldsymbol{A} \right)\boldsymbol{A}_{0}^{-1}\boldsymbol{B}_{0}$, with $a_{n}=\omega_{r}^{2}-\omega_{n}^{2}-i\omega_{n}\eta_{0}.$ The off-diagonal modulation-induced terms are

$$\begin{aligned} {{(A}_{0}^{-1}B_{0})}_{nm}=\left( {\omega_{p0}^{2}}/{a_{n}} \right)\delta_{nm},\#\left( S8 \right) \end{aligned}$$

$$\begin{aligned} ({A_{0}^{-1}\delta B)}_{nm}=\frac{\omega_{p,n-m}^{2}}{a_{n}},n\neq m,\#\left( S9 \right) \end{aligned}$$

$$\begin{aligned} ({-A_{0}^{-1}\left( \delta A \right)A_{0}^{-1}B_{0})}_{nm}=\frac{i\omega_{m}\eta_{n-m}\omega_{p0}^{2}}{a_{n}a_{m}},n\neq m,\#\left( S10 \right) \end{aligned}$$

The static contribution is $\chi_{nn}=\frac{\omega_{p0}^{2}}{a_{n}}$, Under the weak-modulation approximation, the inverse matrix can be expanded to first order as $\chi_{nm}\simeq\frac{\omega_{p,n-m}^{2}}{a_{n}}+\frac{i\omega_{m}\eta_{n-m}\omega_{p0}^{2}}{a_{n}a_{m}},n\neq m$. This expression corresponds to Eq. (9) in the main text. The two terms represent the contributions from plasma-frequency modulation and damping modulation, respectively, providing two distinct Floquet coupling pathways.

**3. FDTD-ADE initial and boundary conditions**

The FDTD–ADE simulations employ an initial-condition excitation,

$$\begin{aligned} E\left( x,0 \right)=Aexp\left[ -\left( \frac{x-x_{0}}{\sigma_{x}} \right)^{2} \right]\exp i\left[ k_{0}\left( x-x_{0} \right)+\theta_{i} \right].\#\left( S11 \right) \end{aligned}$$

where the physical electric field is given by $E_{y}=Re[E\left( x,0 \right)]$. Here, $k_{0}$ denotes the central wavevector, while $\sigma_{x}$ represents the spatial width of the Gaussian envelope and determines the initial wavevector bandwidth. The field amplitude is set to: $A=1 V/m$, and the central wavevector is determined from the unmodulated Lorentz dispersion relation: $k_{0}=Re\left[ \frac{\omega_{0}\sqrt{\varepsilon_{\mathrm{static}}(\omega_{0})}}{c} \right]$, $\varepsilon_{\mathrm{static}}\left( \omega_{0} \right)=\varepsilon_{\infty}+\chi_{static}\left( \omega_{0} \right)=1+\frac{\omega_{p0}^{2}}{\omega_{r}^{2}-\omega_{0}^{2}-i\omega_{0}\eta_{0}}$. The central angular frequency is: $\omega_{0}=2\pi f_{0}=2\pi\times0.3 GHz$, The wave-packet center is initially located at: $x_{0}=6 m$, $\sigma_{x}=\frac{N_{cycle}\lambda_{static}}{4}$, where $N_{cycle}=12$ is a parameter controlling the nominal number of carrier cycles within the wave packet.

The initial magnetic field is specified using the static wave impedance relation $H_{z}=\frac{E_{y}}{Z_{static}}$, where $Z_{static}=\sqrt{\frac{\mu_{0}}{\varepsilon_{0}\varepsilon_{\mathrm{static}}(\omega_{0})}}$. To account for the temporal staggering of the Yee grid, the magnetic field is initialized at $t=-\frac{\Delta t}{2}$. The initial polarization field and its previous time-step value are prescribed as:

$$\begin{aligned} P\left( x,0 \right)=\varepsilon_{0}Re\left[ \chi_{static}E\left( x,0 \right) \right],\#\left( S12 \right) \end{aligned}$$

$$\begin{aligned} P\left( x,-\Delta t \right)=\varepsilon_{0}Re\left[ \chi_{static}E\left( x,0 \right)e^{i\omega\Delta t} \right].\#\left( S13 \right) \end{aligned}$$

The computational domain has a total length of $L=30 m$, with $1.5 m$ thick graded absorbing layers at both boundaries to attenuate outgoing electromagnetic waves. The temporal modulation is spatially uniform and is smoothly switched on and off according to $\omega_{p}^{2}(t)=\omega_{p0}^{2}[1+g(t)m_{p}cos(\Omega t)]$, $\eta(t)=\eta_{0}[1+g(t)m_{\eta}cos(\Omega t+\phi)]$, where $g(t)$ is a temporal envelope controlling the smooth activation, maintenance, and deactivation of the modulation.

Figure S1: initial-condition excitation

Figure S2: Spatial Fourier Spectra of the Incident Electric Field.

**4. Analysis of single-channel modulation and initial excitation phase**

To distinguish the sensitivity to the initial excitation phase and the single-channel modulation phase from the proposed dual-channel relative-phase control of the momentum bandgap, we present FDTD simulations of the electric-field evolution under single-channel modulation with varying initial excitation and modulation phases. Four representative snapshots are selected at the onset of modulation, completion of the turn-on process, onset of the turn-off process, and completion of the turn-off process, approximately labeled as$t=10, 20, 160, 170 ns$.

We introduce an additional phase parameter $\varphi$ for the plasma-frequency modulation, expressed as $\omega_{p}^{2}(t)=\omega_{p0}^{2}[1+g(t)m_{p}cos(\Omega t+\varphi)]$. Meanwhile, $\phi$ remains the modulation phase of the damping factor, given by $\eta(t)=\eta_{0}[1+g(t)m_{\eta}cos(\Omega t+\phi)]$ We set the incident angle as $\theta_{i}=0$.

**(1) Dual-Channel Modulation with Different Initial Excitation Phases**

We consider degenerate excitation at $\omega=\Omega/2$，with simultaneous modulation of the plasma frequency and damping coefficient at modulation depths of $m_{p}=0.3$ and $m_{\eta}=0.5$. Two representative channel relative phases $\phi={\pm\pi}/2$, are examined. As shown in Fig. S3, although the initial excitation phase $\theta$ affects the transient field response, the distinct enhancement and suppression behaviors associated with the two channel relative phases persist across different excitation phases.

Figure S3. Temporal evolution of the spatial electric-field distributions under different modulation phases and incidence angles.

**(2) Electromagnetic Field Evolution under Single-Channel Modulation**

To further clarify the individual contributions of the two modulation channels, we consider plasma-frequency modulation only ($m_{p}=0.3$; $m_{\eta}=0$) and damping modulation only ($m_{p}=0$; $m_{\eta}=0.5$), with the initial excitation phase fixed at $\theta_{i}=0$. we independently vary the plasma-frequency modulation phase $\varphi$ and the damping modulation phase $\phi$, and examine the corresponding cross-sectional electric-field distributions.

As shown in Fig. S4, the two modulation channels exhibit distinct electric-field dynamics. Under the considered conditions, plasma-frequency modulation produces substantially stronger residual electric fields than damping modulation. Moreover, varying the absolute modulation phase leads to differences in the transient field distributions, particularly at later observation times. This phase sensitivity arises from the temporal relationship between the modulation and the incident excitation under finite-duration switching conditions. In contrast, simultaneous modulation of the plasma frequency and damping factor introduces a relative phase that directly modifies the Floquet coupling and the resulting complex-quasifrequency splitting. Thus, the transient phase sensitivity observed under single-channel modulation is physically distinct from the intrinsic Floquet spectral control achieved through dual-channel modulation. The phase-dependent transient responses observed in Fig. S4 arise from the relative timing between the modulation and the prescribed initial excitation. Compared with the dual-channel results, neither single-channel configuration exhibits field amplification under the considered conditions, whereas dual-channel modulation enables pronounced field enhancement through control of the interchannel relative phase.

Figure S4. Temporal evolution of the spatial electric-field distributions under single-channel modulation with different modulation phases.

**5. Phase-dependent field evolution under nondegenerate excitation**

We perform additional FDTD simulations under nondegenerate excitation conditions. The incident frequency is defined as $f_{s}={f_{m}}/2+\Delta f$, where $f_{m}$ is the modulation frequency and $\Delta f$ denotes the frequency detuning from the degeneracy frequency ${f_{m}}/2$. Three detuning conditions are considered: $\Delta f={f_{m}}/6,{f_{m}}/4,{f_{m}}/2$. For each condition, the relative modulation phases $\phi={-\pi}/2$ and $\phi={+\pi}/2$ are compared, while the modulation depths ($m_{p}=0.3$; $m_{\eta}=0.5)$, incident pulse parameters, initial excitation phase, and other numerical settings are kept unchanged.

Since the medium is dispersive, different incident frequencies correspond to different group velocities. Therefore, four representative observation times were selected separately for each excitation frequency. The same observation times were used for both relative modulation phases at each excitation frequency to ensure a consistent comparison.

Figure S5. Phase-dependent electric-field evolution under nondegenerate excitation at $f_{s}={f_{m}}/2+{f_{m}}/6$.

Figure S6. Phase-dependent electric-field evolution under nondegenerate excitation at $f_{s}={f_{m}}/2+{f_{m}}/4$

Figure S7. Phase-dependent electric-field evolution under nondegenerate excitation at $f_{s}={f_{m}}/2+{f_{m}}/2$
